# Prevalence and Factors Associated with Undernutrition among Adults with Major Depressive Disorder in Northwest Ethiopia

**DOI:** 10.1155/2016/7034582

**Published:** 2016-11-21

**Authors:** Edmialem Gezahegn, Melkie Edris, Berihun Assefa Dachew

**Affiliations:** ^1^Psychiatric Clinic, University of Gondar Hospital, Gondar, Ethiopia; ^2^Departmpent of Human Nutrition, Institute of Public Health, University of Gondar, Gondar, Ethiopia; ^3^Department of Epidemiology and Biostatistics, Institute of Public Health, University of Gondar, Gondar, Ethiopia

## Abstract

*Background.* Undernutrition and major depressive disorder are frequently co-occurring. Patients with impaired mental health are strongly vulnerable to the risks of having involuntary weight loss or deficiency of essential nutrients. However, there is no study which assesses undernutrition among major depressive patients in Ethiopia.* Method.* A total of 422 clients were included in the study. Structured questionnaires and anthropometric measurements were used for collecting the data. Bivariate and multivariate logistic regression model was fitted to identify factors associated with undernutrition. Odds ratio with 95% confidence interval was computed to determine the level of significance.* Results.* The prevalence of undernutrition was 31.4% [95% CI: 27.2–36.0]. Being in a rural residence [AOR = 1.84, 95% CI (1.18–2.85)], taking multiple medication [AOR = 1.77, 95% CI (1.03–3.05)], taking prescribed diet [AOR = 1.90, 95% CI (1.06–3.41)], and current use of alcohol [AOR = 2.96, 95% CI (1.34–6.55)] were factors significantly associated with undernutrition among depressive patients.* Conclusion.* The prevalence of undernutrition among adults with major depressive disorder was found to be higher than the general population. Appropriate nutritional education and nutritional assessment are recommended during the course of major depressive disorder.

## 1. Background

Mental health problems account for 13% of the total burden of disease [1, 2]. Malnutrition and major depressive disorder are common health problems [3, 4]. According to World Health Organization (WHO) estimate, major depressive disorder is projected to become the leading cause of disability and the second leading contributor to the global burden of disease by the year 2020 and the impact of this burden of disease is thought to be worse in developing countries [5].

Malnutrition is a major public health problem in developing countries including Ethiopia. According to the 2011 Ethiopian Demographic and Health Survey, the prevalence of moderately/severely malnutrition among Ethiopian men aged 15–59 years was 13.4% and the prevalence is 11.8% (women) and 17% (men) in Amhara region where the study is conducted [6]. It is also the most important risk factor for the burden of many diseases like TB [7], pneumonia [8], visceral leishmaniasis [9], and mental disorders [10].

Malnutrition and major depressive disorder are frequently cooccurring among adults with their interrelated effects and exacerbate one another in a vicious cycle. In developed countries malnourished persons have a 55% increased risk of major depression over time and depressed persons have a 58% increased risk of becoming malnutrition but far less known in developing country [11]. Moreover, impaired mental health is strongly vulnerable to the risks of being involuntary weight gain, weight loss, or deficiency of essential nutrients [12–14]. This is because of poor self-care, being unable to shop or prepare foods, poor balanced diet, and unhealthy lifestyle. In addition, loss of appetite, being unable to eat regularly, and energy expenditure contribute for this association [4, 13].

The sociodemographic characteristics of the respondents (such as sex, age, race/ethnicity, education status, and area of residence), dieting for medical reasons, use of psychiatric medicines, use of alcohol, and use of substances were factors associated with nutritional status of adults with major depressive disorder [12, 15, 16].

Although there are an abundance of research about the underlying conditions that ultimately lead to undernutrition and major depressive disorder individually, little is known about the burden of undernutrition among depressive patients in developing countries [17]. In Ethiopia, many studies have been conducted to identify the magnitude and consequence of undernutrition in different population groups. However, the issue of undernutrition was not well addressed among adults with major depressive disorder. Therefore, assessing the nutritional status of major depressive patients is crucial for identifying the multidimensional conditions where management requires a holistic approach by several methods of assessment and intervention, including nutrition-related health indicators, dietary intake, and energy expenditure [15].

Thus, this study was able to disclose the prevalence and associated factors of undernutrition among adults with major depressive disorder at the University of Gondar Teaching Hospital and Bahir Dar Felege Hiwot Referral Hospital. The findings of this study will be useful for policy makers, program managers, and other concerned bodies to design appropriate strategies.

## 2. Methods and Materials

### 2.1. Study Design and Setting

Institutional based cross-sectional study design was conducted to determine prevalence of undernutrition and associated factors among adults with major depressive disorder. The study was conducted in psychiatry outpatient clinic at University of Gondar (UOG) Teaching Hospital and Bahir Dar Felege Hiwot Referral Hospital. University of Gondar teaching Hospital is located in Gondar town, 738 km way form Addis Ababa, capital city of Ethiopia, to Northwest direction and Felege Hiwot Referral Hospital is found in Bahir Dar town Ethiopia, the capital city of Amhara Regional State, and situated 565 Km away from Addis Ababa to Northwest direction. Approximately, 8,000 adult patients per year have enrolled in chronic care from psychiatry outpatient clinics in both hospitals, of which, a total of 4500 patients per year with major depressive disorder (MDD) have been attending in both outpatient clinics (2,500 from UOG teaching hospital and 2,000 from Felege Hiwot Referral Hospital).

#### 2.1.1. Source and Population

All psychiatry clinic outpatient adults with MDD who attend in the University of Gondar Teaching Hospital and Felege Hiwot Referral Hospital Bahir Dar were the source population for this study.

#### 2.1.2. Inclusion and Exclusion Criteria

All adults with major depressive disorder were included, whereas pregnant and lactating women (<6 months) were excluded from the study.

#### 2.1.3. Sample Size, Sampling Technique, and Sampling Procedures

The sample size of the study was calculated using the formula for the estimation of single proportion with the assumptions of 95% confidence interval (*z*), 5% marginal error (*d*), and taking proportion undernutrition among depressive patients as 50%.

(1) n=z2p1−Pd2,n=1.9620.51−0.50.052=384.

With this assumption, the sample size becomes 384. Considering 10% nonresponse rate the final sample size becomes 422. Proportional allocation is used to maintain proportionality among the two hospitals and then study participants from each hospital were selected by using systematic random sampling methods. Thus, 235 were from UOG Hospital and the rest 188 were from Bahir Dar Felege Hiwot Hospital.

### 2.2. Data Collection Instrument

Data were collected by using interviewer administered structured questionnaire and anthropometric measurements. The questionnaire contains sociodemographic characteristics, clinical factors, dietary-related factors, behavioral factors, and psychosocial factors. The questionnaires were prepared in English and then translated to Amharic (local language) and again back translated to English to check its consistency. Major depressive disorder and sleep disorder were diagnosed based on DSM-IV criteria [18]. Study subjects are considered as current use of alcohol or cigarette smoker, when they use specified substance at least once in the last three months and respondents who were taking ≥4 types of medication per day were considered as taking multiple medications.

### 2.3. Anthropometric Measurements

A studio meter was used to measure the height of the study subjects. Each study subject was asked to stand on the surface, with weight distributed evenly on both feet, heels together against the studio meter, and the head positioned so that the line of vision was perpendicular to the body. The movable headboard was brought to the topmost point on the head with sufficient pressure to compress the hairs. The well-calibrated scale was used to weigh the study participants. To avoid the variability among the data collectors, the same measurement was employed for a given anthropometric measurement more than one time. Finally, BMI was calculated as kg/m^2^ to determine the nutritional status of the respondents. A score less than 18.5 is considered as having undernutrition. It can be further classified as mild (17–18.4), moderate (16–16.9), and severe undernutrition (<16).

### 2.4. Data Processing and Analysis

The data were coded and entered into Epi-Info version 7 and exported to SPSS version 20.0 software packages for analysis. Both descriptive and analytical statistical procedures were utilized. Binary logistic regression was used to identify factors associated with undernutrition among the depressive patients. Variables with a bivariate *P* value < 0.20 were further fitted into multivariate models for controlling the possible effect of confounders, and finally the variables that had significant association with depression were identified on the basis of odds ratio (OR), with 95% confidence interval (CI) and *P* value (*P* < 0.05). The variables were entered to the multivariate model using the Backward Stepwise (Likelihood Ratio) regression method. Model fitness was checked using Hosmer and Lemeshow goodness-of-fit test (*P* = 0.6). Multicollinearity was checked using variance inflation factor (VIF = 1.5–2.5).

### 2.5. Ethical Considerations

Ethical clearance was obtained from Institutional Review Board (IRB) of the University of Gondar. Written informed consent was obtained from respondents who were selected to participate in the study.

## 3. Results

### 3.1. Sociodemographic Characteristics

A total of 405 respondents were included in the study with a response rate of 95.5%. Among those 228 (56.3%) were females. About 40% of the respondents were married and 63% of the respondents were rural dwellers (Table 1).

### 3.2. Clinical Characteristics of Respondents

Nearly half 206 (50.9%) of the respondents were under Amitriptyline medication. About 24% of the respondents were taking multiple (4 or more) medication per day.

From a total of 405 respondents only 98 (24.2%) had known previous psychiatric disorder and 37 (9.1%) of them had known previous medical disorders like TB, pneumonia, malaria, and acute gastritis. Two hundred and twelve (52.3%) of the respondents had no sleep problem (Table 2).

### 3.3. Dietary and Behavioral Characteristics of Respondents

Among respondents 56 (13.8%) of the participants were taking prescribed diet due to different medical reason in their dietary habit. The commonest food eaten by the majority (76.6%) of the respondents was injera (Ethiopian traditional food).

Regarding to their behavioral characteristics 55 (13.6%) participants used alcoholic beverages in the past three months and about 47% of them used greater than once per day (Table 3).

### 3.4. Prevalence of Undernutrition

The overall prevalence of undernutrition among adults with major depression disorder was 31.4% [95% CI: 27.2–36.0] and 23.7% [95% CI: 18.4–28.6] were mild, 4.7% [95% CI: 1.6–7.8] were moderate, and 3.0% [95% CI: 1.2–4.3] were severe undernutrition cases.

### 3.5. Factors Associated with Undernutrition

In multivariate analysis, residence, multiple medication intake, taking prescribed diet, and current use of alcohol were significantly associated with undernutrition among patients with major depressive disorder.

Participants who live in rural areas were nearly two times more likely [AOR = 1.84, 95% CI (1.18–2.85)] to have undernutrition as compared to participants who live in urban areas. On the other hand, participants who have used multiple medications per day were 1.70 times more likely [AOR = 1.70, 95% CI (1.01–2.87)] to have undernutrition as compared to nonusers.

Participants who were not taking the prescribed diet due to medical reasons were nearly two times more likely to have undernutrition than those who had taken prescribed diet [AOR = 1.90, 95% CI (1.06–3.41)]. Moreover, participants who were using alcoholic beverages in the past three months were three times more likely to have undernutrition as compared to participants who had not used [AOR = 2.96, 95% CI (1.34–6.55)] (Table 4).

## 4. Discussion

The prevalence of undernutrition in this study was found to be 31.4% (95% CI: 27.2–36.0). This finding is relatively high as compared to similar cross-sectional studies conducted in Taiwan [13], Portugal [19], and Bangladesh [20], in which 11%, 12%, and 26% of patients with major depressive disorder had undernutrition, respectively.

Moreover, this prevalence was also much higher than the previous study done in Iran (1.43%) [21] and Boston (1.6%) [22]. The difference might be due to socioeconomic difference, difference in dietary habits, and type of medication intake.

The findings of multivariate analysis revealed that the likelihood of being undernutrition was found to be higher among patients who come from rural area as compared to urban [AOR = 1.84, 95% CI (1.18–2.85)]. This finding was in line with the finding reported from Nepal [16]. This could be due to the fact that patients coming from rural area were engaged in high energy expending activities as compared to urban dwellers. Moreover, those study subjects from rural area might not be aware of healthy eating due to poor educational status or might not get the required amount of diet due to low income [21].

The current study, respondents who took multiple medications (≥4 medications per day) were 1.70 times more likely to have undernutrition as compared to those who had not. This finding is supported by the previous studies done in Portugal [19], Boston [22], and Taiwan [13]. This could be due to the fact that medical comorbid illnesses could be associated with increased side effects and treatment resistance related to metabolic dysfunction which in turn leads to undernutrition [19, 22].

Moreover, a depressive patient who was not taking prescribed diet was two times more likely to have undernutrition as compared to those who had taken prescribed diet. This could be due to the fact that patients who had not take prescribed diet might be prone to different diseases and highly predisposed to different comorbidities, which in turn leads to undernutrition [16].

In addition, current use of alcohol was significantly associated with undernutrition among depressive patients. Those participants who used alcoholic beverages were nearly 3 times more likely to have undernutrition as compared to participants who had not drunk in the past three months. This is due to the fact that alcohol use interferes with the absorption and storage micronutrients. In addition, alcohol irritates the gastrointestinal system; it increases acid secretion by the stomach which can injure the lining of the small intestine and interferes with the ability to absorb vital nutrients. Furthermore, alcohol is a toxin that has to be deactivated by the liver, during the detoxification process the body uses thiamin, zinc, and other important nutrients and this can deplete the investments of healthy metabolism and also cause low mood, irritability, and/or aggressive behavior as well as more serious and long-term mental health problems which further predispose to undernutrition [4, 23].

The cross-sectional nature of the study design does not show the temporal relationship. Moreover, recall bias and reporting bias are the potential limitations of this study. However, interviewers allowed sufficient time for adequate recall of long-term memory.

## 5. Conclusion

The prevalence of undernutrition was found to be high in this study. Residences, multiple medications, taking prescribed diet, and current use of alcohol were factors associated with undernutrition among depressive patients. Therefore, prevention as well as early detection and treatment of undernutrition is mandatory for patients with major depressive disorders with special emphasis among patients who come from a rural area and patient taking multiple medications. Furthermore, awareness creation on the prevention of alcohol use among depressive patient is also recommended.

## Figures and Tables

**Table 1:** Sociodemographic characteristics of adults with major depressive disorder (MDD) in University of Gondar and Felege Hiwot Referral Hospital (*n* = 405).

Variables	Frequency	Percent
Sex		
Male	177	43.7
Female	228	56.3
Age in years		
18–26	105	25.9
27–35	112	27.7
36–45	98	24.2
>45	90	22.2
Marital status		
Married	161	39.8
Single	157	38.8
Separate/divorced	60	14.8
Widowed	27	6.7
Living condition		
With family	342	84.4
Alone	63	15.4
Educational status		
Cannot read and write	107	26.4
Primary school	129	31.9
Secondary school	106	26.2
Higher education	63	15.6
Occupation		
No job	126	31.1
Private employee	93	23.0
Governmental employee	56	13.8
Farmer	52	12.8
Merchant	51	12.6
Daily laborers	27	6.7
Monthly income (USD)		
<50	146	36.0
50–100	139	34.3
>100	120	29.6
Residence		
Rural	254	62.7
Urban	151	37.3

**Table 2:** Clinical characteristics of respondents with major depressive disorder in University of Gondar Referral Teaching Hospital and Felege Hiwot Referral Hospital, Northwest Ethiopia, 2015 (*n* = 405).

Variable	Frequency	Percent
Length of treatment for MDD in month		
<3	114	28.1
3–8	101	24.9
9–27	89	22.0
>27	101	24.9
Type of medication for MDD		
Only amitriptyline	206	50.9
Only fluoxetine	146	36.0
Amitriptyline & Chlorpromazine	25	6.2
Fluoxetine & Chlorpromazine	14	3.5
Imipramine or others	14	3.5
Food intake declined		
Yes	170	42
No	230	58
Past psychiatric disorders		
Yes	98	24.2
No	307	75.8
Known past medical disorders		
Yes	37	9.1
No	368	90.9
Known current comorbidity		
Yes	30	7.2
No	375	89.5
Under multiple medication		
Yes	99	24.4
No	306	75.6
Sleep problem		
Yes	122	47.6
No	212	52.3

**Table 3:** Individual characteristics related to dietary and behavioral characteristics of respondents with major depressive disorder in University of Gondar Referral Teaching Hospital and Felege Hiwot Referral Hospital, Northwest Ethiopia, 2015 (*n* = 405).

Variable	Number	Percent
Taking prescribed diet		
Yes	56	13.8
No	349	86.2
Food commonly eaten		
Injera	311	76.6
Bread/Kita	69	17
Qolo (cereals)	25	6.2
Frequency of meal per day		
Two times	135	33.3
Three times	241	59.5
Four times	29	7.2
Taking breakfast		
Yes	253	62.5
No	152	37.5
Fruit & vegetable per day		
Yes	132	32.6
No	273	67.4
Water or fluid intake/day		
<3 litters	282	69.9
3–5 litters	95	23.5
>5 litters	28	6.9
Current smoker		
Yes	33	14.6
No	372	85.4
Current use of alcohol		
Yes	55	13.6
No	350	86.4
Frequency alcohol use		
Once per day	21	38.2
>1 per day	26	47.3
Once per week	8	12.7

**Table 4:** Bivariate and multivariate analysis of factors associated with undernutrition among adults with major depressive disorder at University of Gondar Referral Teaching Hospital & Felege Hiwot Referral Hospital.

Characteristics	Undernutrition		COR (95% CI)	AOR (95%)
	Yes	No		
Sex				
Male	62	115	1	1
Female	65	163	1.35 (0.89–2.06)	1.42 (0.92–2.21)
Living condition				
Alone	25	38	0.65 (0.37–1.13)	0.76 (0.40–1.44)
With family	102	240	1	1
Residence				
Rural	66	188	1.93 (1.26–2.97)^*∗*^	1.84 (1.18–2.85)^*∗*^
Urban	61	90	1	1
Medication for MDD				
Only amitriptyline	63	143	0.66 (0.33–1.32)	0.87 (0.41–1.88)
Only fluoxetine	52	94	0.53 (0.26–1.10)	0.60 (0.28–1.31)
Other	12	41	1	1
Known comorbid disease				
Yes	6	24	1.91 (0.76–4.76)	1.51 (0.50–4.57)
No	121	254	1	1
Taking multiple medication				
Yes	51	119	1.77 (1.03–3.05)^*∗*^	1.70 (1.01–2.87)^*∗*^
No	76	159	1	1
Sleep problem				
Yes	53	140	1.19 (0.96–2.59)	1.5 (0.78–2.50)
No problem	22	49	1	1
Taking prescribed diet				
Yes	25	31	1	1
No	102	247	1.95 (1.09–3.47)^*∗*^	1.09 (1.06–3.41)^*∗*^
Alcohol use				
Yes	8	47	3.03 (1.39–6.61)^*∗*^	2,96 (1.34–6.55)^*∗*^
No	119	231	1	1

^*∗*^Statistically significant at *P* < 0.05.

## Abbreviations

AOR: Adjusted odds ratio
BMI: Body mass index
COR: Crude odds ratio
DSM-IV: Diagnostic and Statistical Manual of Mental Disorders 4th edition
Kg/m^2^: Kilogram per meter square
MDD: Major depressive disorder
OR: Odds ratio
SPSS: Statistical package for the social sciences
WHO: World Health Organization.
